# Alterations of Erythrocytic Phosphorylated Alpha-Synuclein in Different Subtypes and Stages of Parkinson's Disease

**DOI:** 10.3389/fnagi.2021.623977

**Published:** 2021-09-29

**Authors:** Xu-Ying Li, Wei Li, Xin Li, Xu-Ran Li, Linjuan Sun, Weiwei Yang, Yanning Cai, Zhigang Chen, Jun Wu, Chaodong Wang, Shun Yu

**Affiliations:** ^1^Department of Neurobiology, Xuanwu Hospital of Capital Medical University, Beijing, China; ^2^Department of Neurology, Xuanwu Hospital of Capital Medical University, Beijing, China; ^3^Department of Neurology, Xiyuan Hospital, China Academy of Chinese Medical Sciences, Beijing, China; ^4^Department of Neurology, Dongfang Hospital of Beijing University of Chinese Medicine, Beijing, China; ^5^Department of Neurology, Peking University Shenzhen Hospital, Guangdong, China; ^6^National Clinical Research Center for Geriatric Diseases, Beijing, China

**Keywords:** Parkinson's disease, phosphorylation, α-synuclein, red blood cells, biomarker

## Abstract

Serine 129-phosphorylated alpha-synuclein (pS-α-syn) is a major form of α-syn relevant to the pathogenesis of Parkinson's disease (PD), which has been recently detected in red blood cells (RBCs). However, alterations of RBC-derived pS-α-syn (pS-α-syn-RBC) in different subtypes and stages of PD remains to be investigated. In the present study, by using enzyme-linked immunosorbent assay (ELISA) to measure pS-α-syn-RBC, we demonstrated significantly higher levels of pS-α-syn-RBC in PD patients than in healthy controls. pS-α-syn-RBC separated the patients well from the controls, with a sensitivity of 93.39% (95% CI: 90.17–95.81%), a specificity of 93.11% (95% CI: 89.85–95.58%), and an area under the curve (AUC) of 0.96. Considering motor subtypes, the levels of pS-α-syn-RBC were significantly higher in late-onset than young-onset PD (*p* = 0.013) and in those with postural instability and gait difficulty than with tremor-dominant (TD) phenotype (*p* = 0.029). In addition, the levels of pS-α-syn-RBC were also different in non-motor subtypes, which were significantly lower in patients with cognitive impairment (*p* = 0.012) and olfactory loss (*p* = 0.004) than in those without such symptoms. Moreover, the levels of pS-α-syn-RBC in PD patients were positively correlated with disease duration and Hoehn & Yahr stages (H&Y) (*p* for trend =0.02 and <0.001) as well as UPDRS III (*R*^2^ = 0.031, *p* = 0.0042) and MoCA scores (*R*^2^ = 0.048, *p* = 0.0004). The results obtained suggest that pS-α-syn-RBC can be used as a potential biomarker for not only separating PD patients from healthy controls but also predicting the subtypes and stages of PD.

## Introduction

Parkinson's disease (PD) is an age-related neurodegenerative disorder, characterized by akinesia/bradykinesia, rigidity, tremor, and postural instability (Sveinbjornsdottir, 2016). According to motor symptoms, PD can be classified into two major subtypes: tremor-dominant (TD) and postural instability and gait difficulty (PIGD) phenotypes (Stebbins et al., 2013). In addition, PD patients also manifest various non-motor symptoms such as constipation, hyposmia, depression, sleep disorders, and cognitive impairment (Sveinbjornsdottir, 2016). The complexity and variability of the PD symptoms reflects the heterogeneity of its neuropathology between individual patients.

The most characteristic neuropathologic change in PD is the formation of proteinaceous inclusions, known as Lewy bodies (LBs) and Lewy neurites (LNs), which are mainly composed of aggregated alpha-synuclein (α-syn) (Spillantini et al., 1998), a small soluble protein normally enriched in pre-synaptic terminals (Cheng et al., 2011). The presence of α-syn aggregates in Lewy pathology indicates an implication of this protein in the etiology and pathogenesis of PD. The implication of α-syn in PD is further evidenced by genetic studies showing that point mutations and copy number variants in the SNCA gene encoding α-syn are associated with early-onset familial PD (Polymeropoulos et al., 1997; Ahn et al., 2008; Olgiati et al., 2015). According to Braak staging, Lewy pathology processes in a stereotypic, topographic manner in the nervous system. It starts probably in the enteric nervous system and/or olfactory bulb, and then spreads via prion-like propagation to the substantia nigra (SN) and further areas in the central nervous system (CNS), inducing either dysfunction or degeneration of the affected neurons and various motor and non-motor symptoms (Braak and Tredici, 2017; Rietdijk et al., 2017). In addition to continuous progression, the load and distribution of Lewy pathology are varied in different clinical subtypes of PD (Selikhova et al., 2009; Van de Berg et al., 2012).

The close implication of α-syn in the etiology and pathogenesis of PD makes it an ideal biomarker for PD diagnosis. Multiple sources and species of α-syn, including those from the brain, cerebrospinal fluid (CSF), blood, saliva, and the peripheral tissues, have been tested for their utility in diagnosing PD and other synucleinopathies, among which serine 129-phosphorylated α-syn (pS-α-syn) in CSF and plasma is mostly investigated (Atik et al., 2016; Fogue Fayyad et al., 2019; Parnetti et al., 2019), due to its close association with the pathogenesis of PD (Anderson et al., 2006; Oueslati, 2016). All these studies demonstrate an elevation of pS-α-syn in the CSF and plasma of PD patients. However, the diagnostic performance of CSF- and plasma-derived pS-α-syn is low (Foulds et al., 2011, 2013; Wang et al., 2012, 2018; Majbour et al., 2016; Cariulo et al., 2019), possibly due to the presence of interacting factors such as lipid proteins, heterophilic antibodies, and contaminations by platelets and hemolysis (Anderson et al., 2006; Ishii et al., 2015; Atik et al., 2016; Emamzadeh and Allsop, 2017; Fogue Fayyad et al., 2019; Parnetti et al., 2019). Since red blood cells (RBCs) contain a high concentration of α-syn (Barbour et al., 2008), some researchers have turned to study the utility of the RBC-derived α-syn as a PD biomarker. This detection can not only avoid the interfering factors encountered in detecting plasma-derived α-syn, but also could be more stable due to its high concentration. Previous studies have demonstrated positive results for the association between the RBC-derived α-syn and PD. For example, studies by Abd-Elhadi et al. and ourselves provided evidence showing an increase in the levels of RBC-derived total and oligomeric-α-syn with PD (Abd-Elhadi et al., 2015; Wang et al., 2015); some studies also showed elevations of some post-translationally modified (PTM) α-syn (pY125, pS129, nY39, AGE, and SUMO-1) (Vicente Miranda et al., 2017; Tian et al., 2019) and proteinase K-resistant α-syn (Abd-Elhadi et al., 2015; Tian et al., 2019) in the RBCs of PD patients. However, if alterations of pS-α-syn in the RBCs (pS-α-syn-RBC) are associated with the subtypes and stages of PD remains to be elucidated.

In this study, an enzyme-linked immunosorbent assay (ELISA) was used to measure pS-α-syn-RBC, and the associations of pS-α-syn-RBC alterations with various clinical variables, including subtypes, age at onset (AAO), disease duration, Hoehn & Yahr (H&Y) stages, and UPDRS III (Unified Parkinson's disease Rating Scale III) scores were analyzed.

## Materials and Methods

### Study Design and Participants

The PD patients in the study were recruited from Xuanwu Hospital of Capital Medical University, Dongfang Hospital of Beijing University of Chinese Medicine, and Peking University Shenzhen Hospital, from June 2018 to July 2020. During the same period, the healthy control subjects were enrolled from the Physical Examination Centers of Xuanwu Hospital and Xiyuan Hospital, China Academy of Chinese Medical Sciences. Clinical data of both PD patients and healthy controls were first reviewed by investigators from each hospital, and re-evaluated by two senior movement disorder specialists, specifically for the project. All patients were diagnosed based on the MDS clinical diagnostic criteria for PD released in 2015 (Postuma et al., 2015). *De novo* PD patients were identified using the following criteria: (i) disease duration <2 years; (ii) no history of present or past therapy with anti-parkinsonian agents (Mollenhauer et al., 2016). Patients with the following conditions were excluded: (i) Parkinsonian syndromes resulting from cerebrovascular, hypoxic, traumatic, infectious, metabolic, or systemic diseases affecting the CNS; (ii) Parkinson's plus syndromes, including multiple system atrophy (MSA), dementia with Lewy bodies (DLB), progressive supranuclear palsy (PSP), and corticobasal degeneration (CBD); (iii) ambiguous diagnosis due to uncertain clinical or imaging features; (iv) a first degree relative with PD. The control subjects were enrolled fulfilling the following criteria: (1) no motor symptoms (tremor, bradykinesia, restless legs); (2) no history and symptoms of functional constipation and rapid eye movement behavioral disorder (RBD), and less than two other non-motor symptoms, including cognitive decline, olfactory loss, depression, anxiety, according to the cut-off values of each scale for such symptoms; (3) no history and symptoms of stroke, dementia, hypoxia-related disorders (epilepsy, COPD, asthma, etc.); (4) no history and symptoms of blood diseases (anemia, erythremia, etc.). Case and control subjects were matched for age and gender. Participants with incomplete or missing demographic and clinical information were excluded from the study.

The study was approved by the Institutional Review Board and Ethics Committees of the participating hospitals. Written informed consent was obtained from each participant or their legal guardians before inclusion in the study.

### Clinical Assessment and Subtype

All subjects were comprehensively assessed for demographic information and clinical characteristics by the site investigators. The quantitative severity of motor and non-motor symptoms (including cognitive impairment, olfactory loss, sleep, and neuro-psychiatric disturbances) was assessed using scales for each symptom (additional information was given in Supplementary Methods). Movement Disorder Society-sponsored revision of the Unified Parkinson's Disease Rating Scale (MDS-UPDRS) Part III score and H&Y stage were used to assess the severity of motor symptoms. The levodopa equivalent daily dose (LEDD) was calculated as previously described (Rabinak and Nirenberg, 2010). The PD patients were classified into distinct clinical subtypes using cut-offs on the (AAO), and the scores for motor and non-motor symptoms, as described before (Jankovic et al., 1990). Motor subtypes of PD were classified as the TD, PIGD, or mixed (MIX) phenotype, according to the ratio of mean tremor score/mean PIGD score in MDS-UPDRS. Non-motor subtypes were defined by the existence of cognitive impairment, neuropsychiatric symptoms, sleep disorders and olfactory decline, according to cut-off points on the scores quantifying these symptoms: Mini-mental State Examination (MMSE) and Montreal Cognitive Assessment (MoCA) for cognitive impairment, Hyposmia Rating Scale (AHRS) for olfactory loss, Rapid Eye Movement (REM) Sleep Behavior Disorder Questionnaire-Hong Kong (RBDQ-HK) for RBD, Hamilton Depression Scale (HAMD) for depression, and Hamilton Anxiety Scale (HAMA) for anxiety.

### Preparation of RBC Samples

At the time of recruitment, whole blood samples (8 ml) were drawn into EDTA anti-coagulant tubes (1.8 mg per milliliter of blood) from the peripheral vein, let stand for 30 min in a vaccine carrier with ice packs (4–8°C), and then centrifuged at 4°C, 1,500 g for 15 min. The upper and middle layers, containing plasma, and white blood cells, were removed and stored for other uses, and the lower layer, containing RBCs, was washed with Hank's balanced salt solution [HBSS without Ca^2+^ and Mg^2+^, 137.93 mM NaCl, 5.33 mM KCl, 0.34 mM Na_2_HPO_4_, 0.44 mM KH_2_PO_4_, 4.17 mM NaHCO_3_, 5.56 mM D-Glucose (Dextrose), pH 7.2–7.4] for three times. Finally, the isolated RBC samples were collected, aliquoted, and preserved in a freezer (−80°C), and quality-checked every 3 months using fresh samples as the control. Protein concentrations were determined with a bicinchoninic acid (BCA) protein assay kit (Pierce Biotechnology, Rockford, IL, USA) before the assay.

### Western Blot Analysis

Purified wild type α-syn and pS-α-syn proteins were separated by 12.5% sodium dodecyl sulfate-polyacrylamide gel electrophoresis (SDS-PAGE), transferred onto polyvinylidene fluoride (PVDF) membranes (Millipore Corp., Bedford, MA, USA), and blocked for 1 h with 5% non-fat milk in Tris-buffered saline containing 0.05% Tween-20 (TBST). The membranes were probed with either rabbit polyclonal anti-p-α-syn (Ser 129) (sc-135638, Santa Cruz Biotechnology, Santa Cruz, CA, USA) (1:5,000) or 3D5 mouse monoclonal anti-human α-syn antibody (RRID: AB_2315787) (1:10,000) (Yu et al., 2007; Vaccaro et al., 2015). After washing with TBST, the membranes were incubated with horseradish peroxidase-conjugated goat anti-rabbit or goat anti-mouse IgG (1:5,000; Vector Laboratories, Burlingame, CA, USA). Immunoreactive bands were visualized by enhanced chemiluminescence, and measured for densitometry with a Versadoc XL imaging apparatus (Bio-Rad). All experiments were conducted in triplicate. To rule out the possibility for the cross-reaction of the anti-pS-α-syn antibody with other proteins, the RBC lysates were first incubated at 4°C overnight with different concentrations of the 3D5 antibody and pS-α-syn antibody (Santa) conjugated to Protein A Sepharose 4B Fast Flow (P9424, Sigma-Aldrich, St. Louis, MO, USA) separately to pre-absorb the endogenous α-syn. Then, the protein A-antibody-α-syn/pS-α-syn complexes were precipitated, and the supernatants were subjected for Western blotting using a different anti-pS-α-syn antibody (Wako). All experiments were conducted in triplicates.

### Enzyme-Linked Immunosorbent Assay

To establish the ELISA for measuring pS-α-syn concentrations, recombinant human wild type α-syn (WT-α-syn) and pS-α-syn were produced and purified (detailed in Supplementary Methods). The calibration curves were constructed using a series of concentrations of WT-α-syn and pS-α-syn. The assay used an anti-pS-α-syn as the capture and the biotinylated anti-WT-α-syn as the detection antibodies. Correlations between the standardized protein concentrations and the absorbances at 405 nm were analyzed. The lower limit of detection (LLOD) was calculated as the mean absorbance of 20 replicate readings of blank samples (buffer only) plus three standard deviations (SD) (Mathiesen et al., 2018). In addition, the lower limit of quantification (LLOQ) was calculated from the mean absorbance of 24 replicate readings of blank samples plus 10 SD. The accuracy of the assay and reproducibility in the sample matrix were assessed by spike-and-recovery and linearity-of-dilution assessments (detailed in Supplementary Methods). Quality control (QC) samples were used in each tested plate. Intra- and inter-test variations of tested and QC samples were expressed by the coefficient of variation (CVs) and calculated as follows: standard deviation/average concentration × 100% (Li et al., 2020).

For measuring pS-α-syn-RBC concentrations, a 96-well ELISA plate was coated with 100 μL/well of the non-biotinylated anti-pS-α-syn antibody (sc-135638, Santa Cruz Biotechnology, Santa Cruz, CA, USA) in coating buffer (0.1 μg/mL), and incubated overnight at 4°C (Liu et al., 2015). After washing with PBS containing 0.05% Tween-20 (PBST) and blocked with 10% BSA in PBST at 37°C for 2 h, 100 μl of RBC samples were added to each well and incubated at 37°C for 2 h. After washes with PBST, 100 μl/well of biotinylated 3D5 antibody (1 μg/mL) in blocking buffer was added. Plates were incubated for 2 h at 37°C. The wells were washed four times with PBST and incubated for 1 h with 100 μl of ExtrAvidin Alkaline Phosphatase (E-2636, Sigma-Aldrich, St. Louis, MO, USA) diluted 1:5,000 in blocking buffer. Following four washes with PBST, 100 μl of enzyme substrate p-nitrophenyl phosphate (pNPP, N1891, Sigma-Aldrich) was added per well, after which the absorbance was read at 405 nm using a Multiskan MK3 microplate reader (Thermo Scientific, UT, USA). All the samples were assayed blinded to the diagnosis and tested in triplicate within the same assay and on the same day.

### Statistical Analysis

Demographic, clinical characteristics, and α-syn data were compared between groups according to the normality of their distributions. Analyses of variance (ANOVA), followed by *post-hoc* tests, was used for normally distributed data (age, AAO, etc.), and the Kruskal-Wallis test was used for skewed data (disease duration, LEDD, pS-α-syn-RBC levels, etc.) to compare differences among all the studied groups. The Chi-squared or Fisher's exact tests were performed to compare the distribution of categorical variables across groups.

Correlations of clinical variables (e.g., AAO, UPDRS III, MMSE, and MoCA scores) with pS-α-syn-RBC concentrations were examined by uni and multivariate linear regression models, with adjustment for confounding factors (e.g., age, sex, and AAO). Logistic regression models were used to analyze the correlation between the biomarker and PD. Receiver operating characteristic (ROC) curves were constructed and the area under the curve (AUC) was calculated to evaluate the performance of the models. A one-degree-freedom linear term was used for trend analysis of pS-α-syn-RBC alterations across intervals of disease duration and H&Y stages. All statistical analyses were performed using IBM SPSS Statistics® v22.0.0.0 (SPSS Inc., Chicago, IL, 2013), Medcalc (Microsoft), and GraphPad Prism® v6.0 (GraphPad Software Inc., La Jolla, CA, 2009), and *P*-values < 0.05 were regarded as statistically significant.

## Results

Between June 2018 and July 2020, 464 potentially eligible patients were recruited with consent. After clinical and neuroimaging re-evaluation and assurance of blood sample quality, our final analysis included 333 patients and 334 healthy controls. All PD patients were subjected to diagnosing, subtyping and staging analyses. Patients with missing scores for some non-motor symptoms were excluded from the latter analyses (Supplementary Image 1).

Demographic and clinical data for all participants are shown in Table 1. The AAO of the PD patients was 55.8 ± 11.78 years, and the disease duration was 5.03 ± 4.48 years. Most of the PD patients (90.99%) were in H&Y stages 1–3.

**Table 1:** Demographic data of the study subjects.

**Variable**	**Control**	**PD**
Number	334	333
Sex (male/female)	174/160	192/141
Age	60.76 ± 10.99	60.37 ± 10.35
Age at onset	NA	55.28 ± 11.78
Education, years	–	9.95 ± 4.82
HandY stage	NA	2 (2, 3)
UPDRS III	NA	26.33 ± 12.70
**Disease duration, years**		
All	NA	5.03 ± 4.48
0–2	NA	1.44 ± 0.58
3–5	NA	3.68 ± 0.99
6–10	NA	7.77 ± 1.71
≥70	NA	13.78 ± 5.86
**Medication**		
Levodopa treatment, *n* (%)	NA	203 (60.96%)
LEDD, mg/day	NA	367.74 ± 382.04
**Severity of non-motor signs**		
MoCA scores	–	23.45 ± 4.28 (*n =* 250)
MMSE scores	–	26.26 ± 3.46 (*n =* 201)
RBDQ-HK scores	–	21.21 ± 18.02 (*n =* 204)
AHRS scores	–	20.41 ± 6.40 (*n =* 195)
HAMD scores	–	10.25 ± 6.58 (*n =* 184)
HAMA scores	–	9.85 ± 5.76 (*n =* 226)

*All data are presented as Mean ± S.D. PD, Parkinson's disease; MSA, Multiple system atrophy; LEDD, Levodopa equivalent daily dose; UPDRS III, Unified Parkinson's Disease Rating Scale part III (motor) score; HandY stage, Hoehn and Yahr stage; MoCA, Montreal Cognitive Assessment; MMSE, Mini-Mental State Examination; RBDQ-HK, Rapid eye movement (REM) sleep behavior disorder questionnaire-Hong Kong; HAMD, Hamilton Depression Scale; HAMA, Hamilton Anxiety Scale; Definition of abnormal: for cognitive decline, MoCA score cut-offs of 17 (illiteracy), 20 (primary school education), 23 (secondary school education) of 30 points; for RBD, a cut-off of 19 on the RBDQ-HK score; for olfactory loss, a cut-off of 22 on the AHRS score; for depression, a cut-off of 8 on the HAMD score; for anxiety, a cut-off of 7 on the HAMA score. NA, not applicable; –, not available*.

The specificity, accuracy, and reproducibility of the ELISA were evaluated using multiple assessments. As shown in our previous study (Li et al., 2020), after production and purification, Coomassie brilliant blue (CBB) staining confirmed a high purity of both the recombinant WT-α-syn and pS-α-syn proteins. In western blotting, the 3D5 anti-α-syn antibody specifically detected WT-α-syn (17 kDa). In contrast, the anti-pS-α-syn antibody did not bind WT-α-syn, but bound purified pS-α-syn (55 kDa) in a concentration-dependent manner. In the detection of RBC lysates using western blot, the anti-pS-α-syn antibody revealed several bands, including three major bands ranging from above 55 to 130 kDa and a very weak band at around 28 kDa. The three major bands were absorbed by pre-incubation of the lysates with overdose of the 3D5 anti-α-syn (Li et al., 2020) and pS-α-syn antibody (Supplementary Image 2) with the 28 kDa weak band remained unaffected, indicating that the pS-α-syn antibody mainly detects the pS-α-syn, which was in an aggregated form. Sine hemoglobin has peroxidase activity, the 28 kDa band may be the hemoglobin dimer, as revealed by the electrochemiluminescence (ECL) reaction (Abd-Elhadi et al., 2015). In the ELISA, the absorbances measured at 405 nm were positively correlated with the standardized pS-α-syn concentrations, with an *R*^2^-value of 0.995 (Li et al., 2020). Further, using the blank samples, the LLOD and LLOQ of the assay were determined to be 0.18 and 0.60 μg/ml, respectively. Spike-and-recovery assessment showed that the recovery rates of pS-α-syn in RBC lysates at low, median, and high spike levels were 87.7, 90.7, and 95.9%, respectively (Supplementary Tables 1–4).

**Figure 1:**
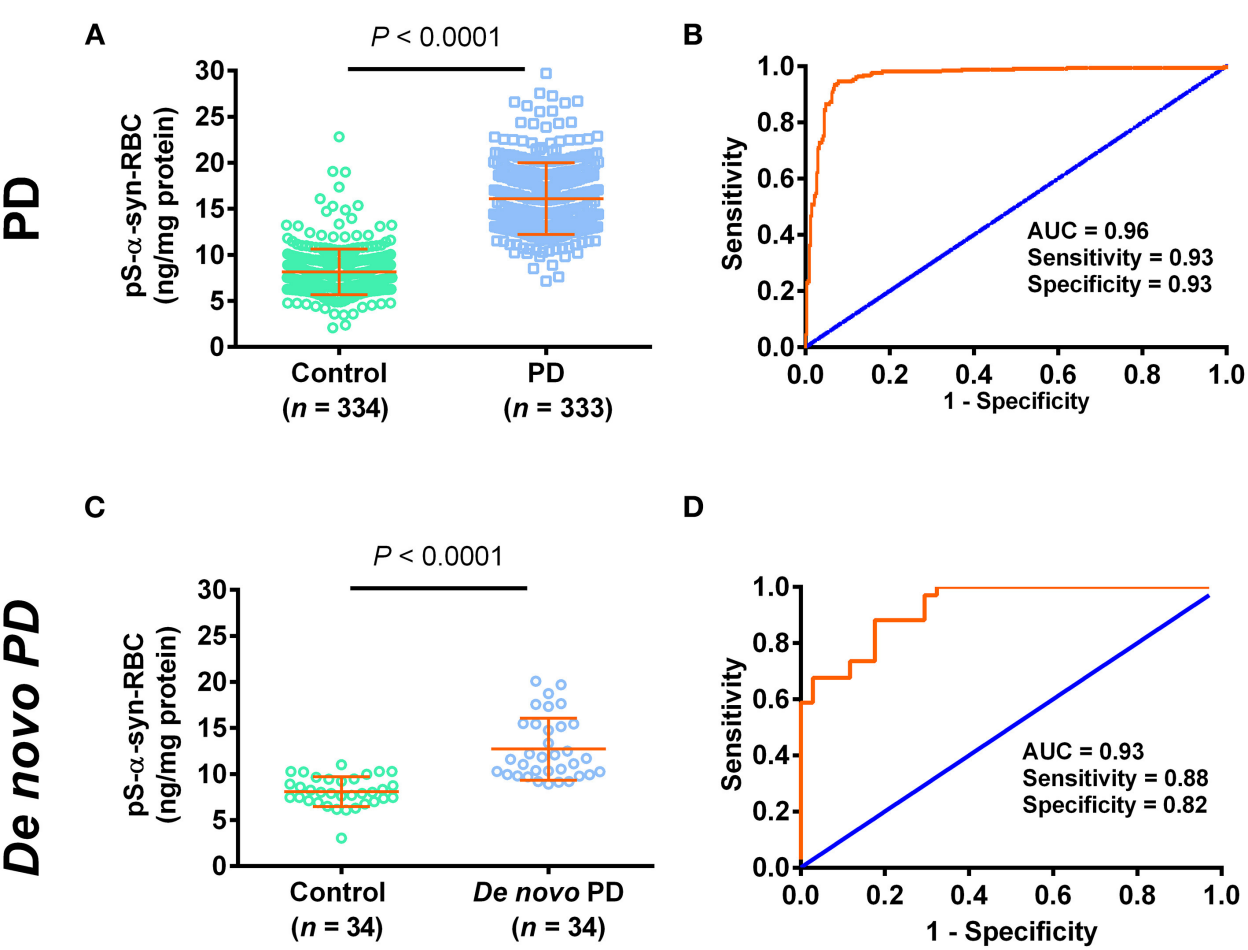
Performance of pS-α-syn-RBC in diagnosing Parkinson's disease. **(A,C)** Concentrations of pS-α-syn-RBC in Parkinson's disease (PD) vs. controls in the PD (PD: 333, control: 334) and *de novo* (PD: 34, control: 34) study. **(B,D)** Receiver operating characteristic (ROC) curves for the diagnosis of PD in the PD and *de novo* PD study.

We then used the ELISA to measure the levels of pS-α-syn-RBC. We showed that the levels of pS-α-syn-RBC were significantly higher in PD patients (14.17 ± 3.58 ng/mg) than in controls (7.22 ± 2.19 ng/mg; *p* < 0.0001). pS-α-syn-RBC separated the PD patients well from the controls, with a sensitivity of 93.39% (95% CI: 90.17–95.81%), a specificity of 93.11% (95% CI: 89.85–95.58%), and an AUC of 0.96 (Figures 1A,B). In a sub-analysis, pS-α-syn-RBC levels in the *de novo* PD patients (*n* = 34; 12.70 ± 3.36 ng/mg) were significantly higher than the age- and sex-matched controls (*n* = 34; 8.10 ± 1.62 ng/mg; *p* < 0.0001) (Figures 1C,D).

**Figure 2:**
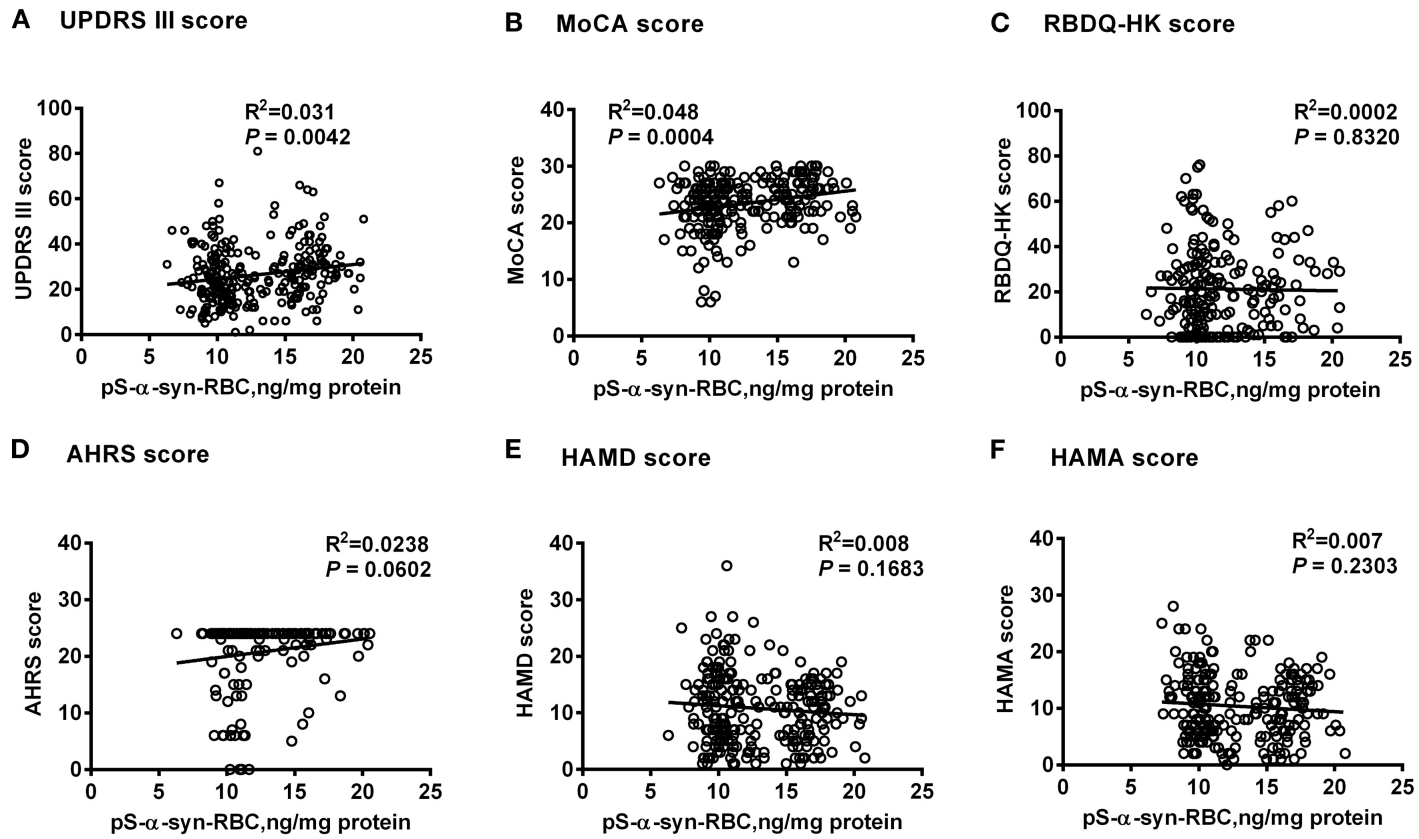
Correlations between pS-α-syn-RBC and scores for motor and non-motor symptoms of Parkinson's disease. Correlations between pS-α-syn-RBC levels and scores for motor and non-motor symptoms were investigated using multivariate linear regression model adjusting age, sex, and disease duration. pS-α-syn-RBC, phosphorylated α-syn in red blood cells; **(A)** UPDRS III, Unified Parkinson's disease Rating Scale part III (motor); **(B)** MoCA, Montreal Cognitive Assessment; **(C)** RBDQ-HK, Rapid eye movement (REM) sleep behavior disorder questionnaire-Hong Kong; **(D)** AHRS, Hyposmia rating scale; **(E)** HAMD, Hamilton Depression Scale; **(F)** HAMA, Hamilton Anxiety Scale.

Multivariate linear regression analysis, adjusting for age, sex, and disease duration, showed that pS-α-syn-RBC levels in the PD patients were positively correlated with UPDRS III scores (*R*^2^ = 0.031, *p* = 0.0042) and MoCA scores (*R*^2^ = 0.048, *p* = 0.0004), but not with RBDQ-HK, AHRS, HAMD, or HAMA scores (*p* > 0.05, Figure 2). Considering the motor subtypes, the pS-α-syn-RBC levels were significantly higher in late-onset PD (LOPD) (*n* = 288; 14.57 ± 4.92 ng/mg) than in young-onset PD (YOPD) (*n* = 45; 14.06 ± 4.82 ng/mg; *p* = 0.013) as well as in patients with the PIGD (*n* = 103; 14.90 ± 5.79 ng/mg) than with the TD subtypes (*n* = 50; 13.05 ± 2.64 ng/mg; *p* = 0.029; Table 2). With respect to the non-motor subtypes, the pS-α-syn-RBC levels in patients with cognitive impairment and olfactory loss were significantly lower than those without such symptoms (*p* = 0.012 and 0.004, respectively; adjusting for age, sex, LEDD, and disease duration; Table 3 and Supplementary Table 5). Moreover, the discrepancies in the level of pS-α-syn-RBC between motor and non-motor subtypes were associated with the AAO. Significant difference between the TD and PIGD subtypes was only observed in patients with the AAO <50 years (*p* = 0.021, adjusting for age, sex, disease duration, and LEDD), while between the cognitively normal and impaired PD patients was only found among those with the AAO between 60 and 69 years (*p* = 0.004; Tables 2, 3; Supplementary Image 3).

**Table 2:** Overall and clinical variable-stratified comparisons of pS-a-syn-RBC levels between motor subtypes of Parkinson's disease.

**Scale**	**All PD (*n =* 333)**	**TD (*n =* 50)**	**PIGD (*n =* 103)**	**MIX (*n =* 180)**	***p* (TD vs. PIGD)**	***p* (PIGD vs. MIX)**	***p* (TD vs. MIX)**
Sex (male/female)	192/141	20/30	64/39	108/72	**0.015**	0.800	**0.015**
Age	60.37 ± 10.35	62.54 ± 11.06	59.48 ± 10.63	60.13 ± 10.73	0.099	0.627	0.162
Age at onset	55.28 ± 11.78	58.13 ± 11.20	54.88 ± 11.34	53.69 ± 14.36	0.149	0.461	**0.034**
Disease duration, y	5.03 ± 4.48	4.42 ± 3.91	4.60 ± 3.24	5.06 ± 4.68	0.798	0.369	0.332
LEDD, mg/day	367.74 ± 382.04	318.03 ± 374.02	397.64 ± 373.56	371.85 ± 388.92	0.369	0.970	0.356
H and Y stage	2 (2, 3)	2 (2, 3)	2 (2, 3)	2 (1, 3)	0.833	0.628	0.339
pS-α-syn-RBC levels	2.31 ± 0.70	2.19 ± 0.43	2.68 ± 1.11	2.21 ± 0.51	**0.001**	**<0.001**	0.855
(raw data, μg/ml)							
pS-α-syn-RBC levels	14.17 ± 3.58	13.05 ± 2.64	14.90 ± 5.79	14.56 ± 4.77	**0.029**	0.573	0.055
(normalized, μg/mg)							
**pS-α-syn-RBC levels in patients stratified by age at ONSET, ng/mg^a^**							
<50	14.06 ± 4.82	11.47 ± 1.75	14.25 ± 5.75	14.40 ± 4.49	**0.021**	0.888	**0.017**
≥50	14.57 ± 4.92	12.18 ± 2.45	14.89 ± 5.79	14.56 ± 4.77	0.101	0.551	0.191
50–59	14.75 ± 5.44	12.33 ± 2.48	16.48 ± 7.07	14.14 ± 4.84	0.087	0.073	0.669
60–69	14.70 ± 4.53	12.21 ± 2.81	13.68 ± 3.75	15.60 ± 5.07	0.894	0.104	0.149
≥70	13.65 ± 4.21	11.76 ± 1.55	15.07 ± 5.56	12.99 ± 3.89	0.311	0.181	0.959
**pS-α-syn-RBC levels in patients stratified by disease duration, ng/mg^b^**							
0–2	13.11 ± 4.21	12.61 ± 2.72	13.74 ± 4.58	12.73 ± 4.44	0.588	0.557	0.929
3–5	13.10 ± 4.37	11.92 ± 2.38	13.91 ± 5.51	13.06 ± 3.95	0.176	0.380	0.431
5–10	13.68 ± 5.03	11.06 ± 0.96	13.48 ± 5.83	14.57 ± 4.88	0.411	0.259	0.092
>10	15.58 ± 4.97	12.30 ± 2.46	17.42 ± 6.68	15.68 ± 4.54	0.146	0.120	0.637
**pS-α-syn-RBC levels in patients stratified by HandY STAGES, ng/mg^c^**							
1	11.44 ± 3.36	10.90 ± 0.92	10.96 ± 3.27	11.84 ± 3.88	0.858	0.647	0.551
2	13.16 ± 4.03	12.01 ± 2.33	12.97 ± 4.39	13.81 ± 4.19	0.508	0.284	0.126
3	14.09 ± 4.95	12.56 ± 2.76	15.01 ± 5.73	14.01 ± 4.86	0.091	0.319	0.256
4–5	16.40 ± 6.76	12.21 ± 2.81	20.87 ± 7.84	15.39 ± 5.25	0.106	**0.050**	0.307

a,b,c *Covariance analysis adjusting for sex, age, LEDD and disease duration; Bold: p < 0.05. YOPD, Young onset Parkinson's disease; LOPD, Late onset Parkinson's disease; TD, Tremor-dominant subtype; PIGD, postural instability and gait difficulty subtype*.

**Table 3:** Overall and clinical variable-stratified comparisons of pS-a-syn-RBC levels between non-motor subtypes of Parkinson's disease.

**Scale**	**Cognitive decline**			**RBD**			**Olfactory loss**		
	**No (*n =* 184)**	**Yes (*n =* 66)**	***p*-value**	**No (*n =* 104)**	**Yes (*n =* 100)**	***p*-value**	**No (*n =* 139)**	**Yes (*n =* 56)**	***p*-value**
Sex	108/76	30/36	–	99/45	45/55	–	74/65	25/31	–
(male/female)									
Age	59.22 ± 10.68	64.12 ± 8.55	–	60.28 ± 11.03	63.41 ± 8.41	–	60.91 ± 10.43	63.36 ± 8.56	–
Age at onset	55.10 ± 11.99	58.75 ± 10.77	–	52.06 ± 18.20	58.41 ± 10.03	–	56.06 ± 12.63	57.78 ± 10.13	–
Disease duration, y	4.73 ± 4.12	4.85 ± 4.82	0.969	4.80 ± 4.52	5.08 ± 4.43	0.180	4.46 ± 4.23	5.66 ± 4.58	0.325
LEDD, mg/day	425.51 ± 424.51	377.12 ± 379.36	0.334	365.84 ± 415.33	394.71 ± 345.73	0.456	360.41 ± 399.05	393.31 ± 319.44	0.612
HandY stage	2 (2, 3)	2 (2, 3)	–	2 (2, 3)	2 (2, 3)	–	2 (2, 3)	2 (2, 3)	–
pS-α-syn-RBC	12.91 ± 3.43	11.64 ± 3.10	**0.012**	11.96 ± 3.23	11.42 ± 2.96	0.101	12.02 ± 3.18	10.74 ± 2.37	**0.004**
**pS-α-syn-RBC levels in patients stratified by age AT ONSET, ng/mg^a^**									
<50	12.58 ± 3.52	12.63 ± 3.07	0.844	11.09 ± 2.32	11.65 ± 3.26	0.866	11.01 ± 2.58	10.56 ± 2.31	0.660
50–59	12.81 ± 3.40	11.16 ± 2.80	0.095	11.59 ± 2.95	11.50 ± 2.96	0.980	12.06 ± 3.15	11.20 ± 2.31	0.288
60–69	13.18 ± 3.45	10.86 ± 2.52	**0.004**	12.04 ± 2.88	12.46 ± 3.31	0.907	12.73 ± 3.43	11.53 ± 2.16	0.477
≥70	11.55 ± 3.14	12.39 ± 4.01	0.137	12.42 ± 3.87	11.43 ± 2.92	0.347	11.66 ± 3.30	10.53 ± 4.11	0.249
**pS-α-syn-RBC levels in patients stratified by disease duration, ng/mg^b^**									
0–2	12.62 ± 3.56	12.72 ± 3.54	0.973	11.77 ± 3.18	12.36 ± 3.53	0.414	12.63 ± 3.46	11.43 ± 2.89	0.281
3–5	12.87 ± 3.56	11.74 ± 2.75	0.565	11.92 ± 3.06	11.54 ± 2.86	0.199	12.15 ± 3.20	11.05 ± 2.20	0.121
5–10	12.77 ± 3.13	10.38 ± 2.48	**0.038**	11.18 ± 2.36	11.27 ± 2.58	0.997	10.89 ± 2.64	10.30 ± 1.88	0.949
>10	14.40 ± 2.95	10.42 ± 2.79	**0.038**	11.16 ± 1.89	13.00 ± 3.66	0.130	11.10 ± 1.84	14.16 ± 2.29	0.137
**pS-α-syn-RBC levels in patients stratified by HandY stages, ng/mg^c^**									
1	12.61 ± 3.17	11.62 ± 3.13	0.244	11.62 ± 3.28	12.15 ± 3.76	0.935	12.26 ± 3.51	11.74 ± 2.91	0.690
2	13.34 ± 3.15	11.66 ± 3.15	**0.002**	11.97 ± 2.91	11.39 ± 2.50	0.153	11.76 ± 2.83	11.43 ± 2.50	0.067
3	13.14 ± 3.74	10.78 ± 2.33	**0.002**	11.19 ± 2.64	12.44 ± 3.66	0.374	12.38 ± 3.63	11.31 ± 2.14	0.463
4–5	15.50 ± 4.03	11.88 ± 2.99	0.504	11.44 ± 2.60	12.72 ± 3.05	0.807	10.14 ± 0.03	10.72 ± 3.05	0.765

a,c *Covariance analysis adjusting for sex, disease duration, and LEDD*;

b *Covariance analysis adjusting for sex, age and LEDD. Definition of abnormal: for cognitive decline, MoCA score cut-offs of 17 (illiteracy), 20 (primary school education), 23 (secondary school education) of 30 points; for RBD, a cut-off of 19 on the RBDQ-HK score; for olfactory loss, a cut-off of 22 on the AHRS score. Bold values: p < 0.05*.

**Figure 3:**
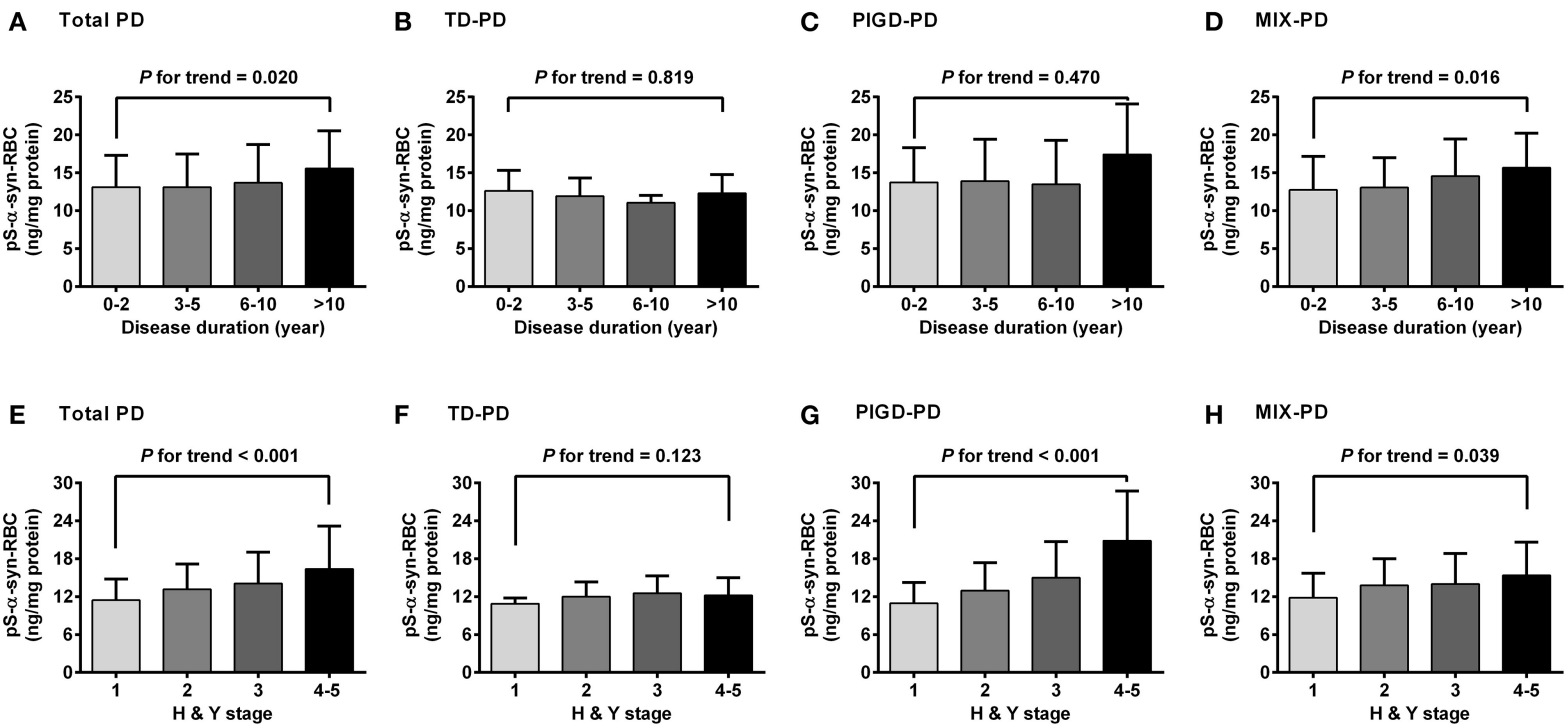
Correlations of pS-α-syn-RBC with disease duration **(A–D)** and HandY stages **(E–H)** of Parkinson's disease and its motor subtypes. Trend was analyzed by using one-degree-freedom linear term. TD-PD, Parkinson's disease (PD) subtype with tremor dominant phenotype; PIGD-PD, PD subtype with postural instability and gait difficulty (PIGD)-dominant phenotype; MIX-PD, PD subtype with intermediate phenotype between TD and PIGD.

**Figure 4:**
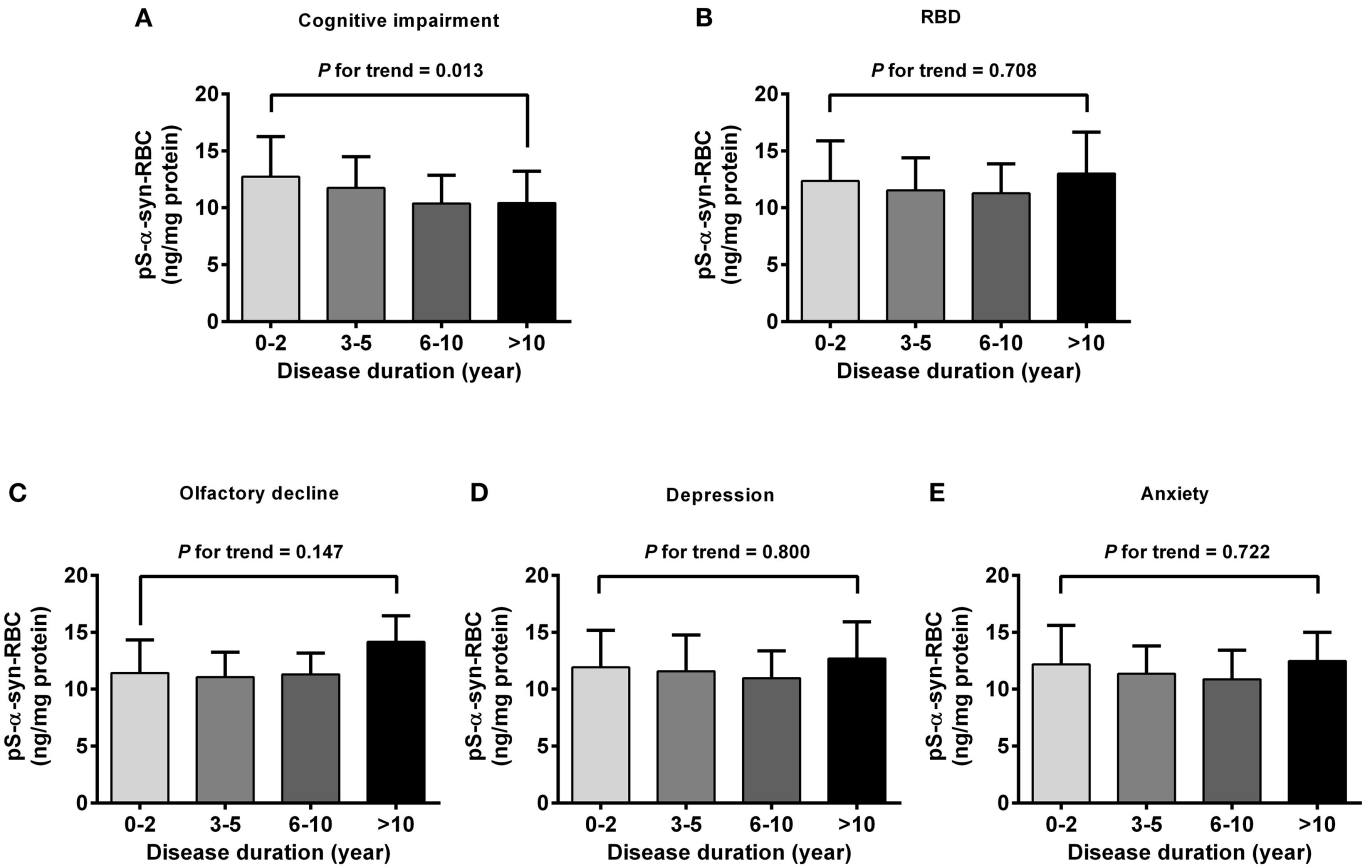
Correlations of pS-α-syn-RBC with disease duration in PD patients with different non-motor subtypes. Correlations between pS-α-syn-RBC and intervals of disease duration in PD patients with non-motor subtypes was analyzed by using one-degree-freedom linear term for trend analysis. **(A)** Cognitive impairment; **(B)** RBD; **(C)** olfactory loss; **(D)** depression; **(E)** anxiety. *p*-values for the changing trend of pS-α-syn-RBC levels across increased disease durations were calculated.

Regarding the association of pS-α-syn-RBC with disease duration and H&Y stage of PD, pS-α-syn-RBC levels were progressively elevated with increasing disease duration (13.11 ± 4.21 ng/mg for 0–2 years; 13.10 ± 4.37 ng/mg for 3–5 years; 13.68 ± 5.03 ng/mg for 6–10 years, and 15.58 ± 4.97 ng/mg for >10 years; *p* for trend =0.020; Figure 3A) and advancing H&Y stages (11.44 ± 3.36 for stage 1, 13.16 ± 4.03 for stage 2, 14.09 ± 4.95 for stage 3, 16.40 ± 6.76 for stage 4–5; *p* for trend <0.001; Figure 3E). Concerning the motor subtypes, we found that higher pS-α-syn-RBC levels were associated with advancing H&Y stages in PIGD-PD (*p* < 0.001; Figure 3G) and increasing disease duration in MIX-PD patients (*p* = 0.016; Figure 3D), but not in TD-PD (*p* > 0.05; Figures 3B,F). With respect to the non-motor subtypes, pS-α-syn-RBC levels decreased with increasing disease duration in patients with cognitive impairment (*p* for trend =0.013), but not with other symptoms (RBD, olfactory dysfunction, depression, or anxiety; Figure 4).

## Discussion

The ELISA assay used for measuring pS-α-syn-RBC levels was established previously and has been applied to detect pS-α-syn in monkey brains (Liu et al., 2015) and pS-α-syn formed in PD plasma (Wang et al., 2016). The detection antibody was 3D5 mouse monoclonal anti-α-syn, which recognizes a sequence of 115–121 amino acids specific to human α-syn (Yu et al., 2007) and has been well-characterized previously for its specificity in detecting α-syn in human RBCs (Wang et al., 2015). The capture antibody was a rabbit polyclonal anti-pS-α-syn. The specificity of this anti-pS-α-syn in detecting pS-α-syn in human RBCs was confirmed by immunodepletion experiments, in which the immunoreactive signals revealed by this antibody in RBC lysates were disappeared by pre-incubating the lysates with the 3D5 anti-α-syn antibody and a different anti-pS-α-syn antibody. In order to further characterize our ELISA assay, we performed the spike-and-recovery and linearity-of-dilution assessments as well as LLOD and LLOQ analyses. The results, together with those from the immunodepletion experiments, suggested an enough sensitivity, specificity, accuracy, and reproducibility of this assay in detecting pS-α-syn in human RBCs.

As blood samples are more accessible and less invasive to obtain, detection of blood-derived α-syn as a biomarker is clinically preferable. Most previous studies have focused on detecting α-syn in blood plasma, which have yielded inconsistent results. Increased (Lee et al., 2006; Duran et al., 2010; Lin et al., 2018), decreased (Li et al., 2007; Ishii et al., 2015), and unchanged (Shi et al., 2010; Foulds et al., 2011; Goldman et al., 2018) levels of total plasma α-syn have been reported in PD patients. Although oligomeric α-syn and pS-α-syn levels in PD plasma tended to increase, their diagnostic values were low (Atik et al., 2016; Fogue Fayyad et al., 2019; Parnetti et al., 2019). The inconsistency and low diagnostic power in detecting plasma α-syn have been attributed to the presence of various interfering factors in the plasma such as lipoproteins, heterophilic antibodies, and contaminations by platelets and hemolysis (Anderson et al., 2006; Ishii et al., 2015; Atik et al., 2016; Emamzadeh and Allsop, 2017; Fogue Fayyad et al., 2019; Parnetti et al., 2019). Because RBCs contain abundant α-syn and the detection of RBC α-syn can avoid the interference encountered in the detection of plasma α-syn, it is believed that the RBC α-syn can be used as an alternative potential biomarker predicting the neuropathological changes of PD. Our present results showed that the levels of pS-α-syn-RBC were significantly higher in the PD patients than in the healthy controls. When this biomarker was used to diagnose PD, it produced a 93.39% sensitivity and a 93.11% specificity, with an AUC as high as 0.96. These values are much better than any reported values obtained in detecting plasma α-syn as well as other species of the RBC α-syn, including total α-syn, oligomeric α-syn, proteinase K-resistant α-syn, and some other PTMs (Glycation, SUMO-1, pY125, and nY39) (Abd-Elhadi et al., 2015; Vicente Miranda et al., 2017; Tian et al., 2019). The high performance of pS-α-syn-RBC in PD diagnosis can be explained not only by the high concentration of pS-α-syn in the RBCs and the lack of the interfering factors existent in the plasma, but also by the close relevance of pS-α-syn to the neuropathology of PD. Our results for the elevation of pS-α-syn-RBC in PD patients is supported by a recently published paper, which showed that erythrocytic pS-α-syn levels in PD patients were increased by more than nine times in comparison to the control values, although the AUC was only 0.71, possibly due to the higher degree of overlap of the measured pS-α-syn values between PD and controls (Tian et al., 2019).

Previous studies have shown that the neuropathological progression and total α-syn levels in plasma and CSF are different between PD subtypes (Selikhova et al., 2009; Van de Berg et al., 2012; Ding et al., 2017; Goldman et al., 2018). Since the levels of pS-α-syn-RBC were significantly increased in PD patients, we questioned if alterations of pS-α-syn-RBC are also different between PD subtypes. We showed that the levels of pS-α-syn-RBC were higher in the PIGD than in the TD subtypes. This discrepancy is in agreement with previous observations that patients with the PIGD subtype have more severe motor disorders, more rapid progression, and more frequent non-motor symptoms than patients with the TD subtype (Jankovic et al., 1990; Van der Heeden et al., 2016; Wu et al., 2016), suggesting a role of pS-α-syn in the motor phenotype and severity of PD. However, how pS-α-syn in the RBCs is correlated with that in the CNS is unclear. Evidence has accumulated that α-syn can be secreted from neuronal cells possibly by exocytosis and transported across the blood-brain barrier (BBB) bi-directionally, blood-to-brain and brain-to-blood. Therefore, it is possible that the brain-derived pS-α-syn can be released into the plasma, where it is further taken up by the RBCs in an unknown mechanism (Matsumoto et al., 2017).

Although PD patients with cognitive impairment have more severe neocortical Lewy body scores and higher plasma α-syn levels than those without cognitive impairment (Selikhova et al., 2009; Van de Berg et al., 2012; Ding et al., 2017; Goldman et al., 2018), we did not find an increase in the level of pS-α-syn-RBC in the cognitively impaired patients. Conversely, the cognitively impaired patients had lower levels of pS-α-syn-RBC in comparison to the cognitively normal patients. Because the PD patients with worse olfaction were more likely to have cognitive impairment (Fullard et al., 2016), it is reasonable that the patients with olfactory loss also had lower levels of pS-α-syn-RBC compared to those with normal olfactory function. The mechanism is not clear. It has been demonstrated that PD patients with cognitive impairment and olfactory dysfunction are associated with increased Aβ and tau pathology (Fujishiro et al., 2008; Irwin et al., 2013), and in the RBCs of PD patients, these proteins were found to interact with α-syn to form the α-syn-Aβ or the α-syn-Aβ heterocomplex (Daniele et al., 2018a,b). Therefore, the lower levels of pS-α-syn-RBC detected in the patients with cognitive impairment and olfactory loss may not be due to the actual reduction of pS-α-syn-RBC but rather to its interaction with Aβ and tau, which affects its accurate detection.

The present study also revealed that higher pS-α-syn-RBC levels were associated with worse motor symptoms, as indicated by UPDRS III scores. Moreover, the levels of pS-α-syn-RBC were positively correlated with disease duration and H&Y stages. These results are in agreement with previous studies showing that dopaminergic neuronal loss is positively correlated with total α-syn burden and disease duration (Dijkstra et al., 2014), suggesting that alterations of the pS-α-syn-RBC level may reflect the neuropathological conditions in PD.

Age at onset (AAO) is an important factor that affects the progression, severity, and phenotype of PD. We found that the levels of pS-α-syn-RBC in LOPD were significantly higher than those in YOPD. This is in line with a recent prospective study showing that an older AAO was associated with a more severe PD phenotype and a greater impairment of dopaminergic function (Pagano et al., 2016). In addition, we found that the differences in the level of pS-α-syn-RBC between the patients with the TD and PIGD subtypes and between the patients with and without cognitive and olfactory impairments were also affected by the AAO. However, with the increase in the AAO, the differences were disappeared, indicating that the neuropathological changes of PD tend to be homogenized with the increase in the AAO.

In conclusion, our study demonstrates that pS-α-syn-RBC levels are increased in PD patients, which separate PD patients from healthy controls, differ in different subtypes and stages of PD, and may reflect the heterogeneity and progression of the neuropathological changes of PD.

## Data Availability Statement

The original contributions presented in the study are included in the article/Supplementary Materials, further inquiries can be directed to the corresponding authors.

## Ethics Statement

The studies involving human participants were reviewed and approved by Xuanwu Hospital of Capital Medical University, Dongfang Hospital of Beijing University of Chinese Medicine, Peking University Shenzhen Hospital and Xiyuan Hospital, China Academy of Chinese Medical Sciences. The patients/participants provided their written informed consent to participate in this study.

## Author Contributions

SY and CW: had full access to all of the data in the study and takes responsibility for the integrity of the data, the accuracy of the data analysis, concept, design, obtained funding, and supervision. All authors: acquisition, analysis, or interpretation of data. CW, SY, and X-YL: drafting of the manuscript. LS, ZC, and JW: critical reversion of the manuscript for important intellectual content. CW and X-YL: experiments performance and statistical analysis. WL, XL, X-RL, WY, YC, LS, ZC, and JW: technical and material support.

## Funding

This work was supported by grants from National Natural Science Foundation of China (NSFC) (82071428 and 81671244) to SY, grants from Ministry of Science and Technology of the People's Republic of China (MOST) (Key Project No. 2016YFC1306000), National Natural Science Foundation of China (NSFC) (No. 81771212), and Special Fund from the Key laboratory of Neurodegenerative Diseases, Ministry of Education of China (PXM2019_026283_000002) to CW.

## Conflict of Interest

The authors declare that the research was conducted in the absence of any commercial or financial relationships that could be construed as a potential conflict of interest.

## Publisher's Note

All claims expressed in this article are solely those of the authors and do not necessarily represent those of their affiliated organizations, or those of the publisher, the editors and the reviewers. Any product that may be evaluated in this article, or claim that may be made by its manufacturer, is not guaranteed or endorsed by the publisher.
