# Supplementary figures and images for: Alterations of Erythrocytic Phosphorylated Alpha-Synuclein in Different Subtypes and Stages of Parkinson's Disease
Source: Front Aging Neurosci. 2021 Sep 29;13:623977. doi: 10.3389/fnagi.2021.623977 (PMC8511781; doi:10.3389/fnagi.2021.623977)

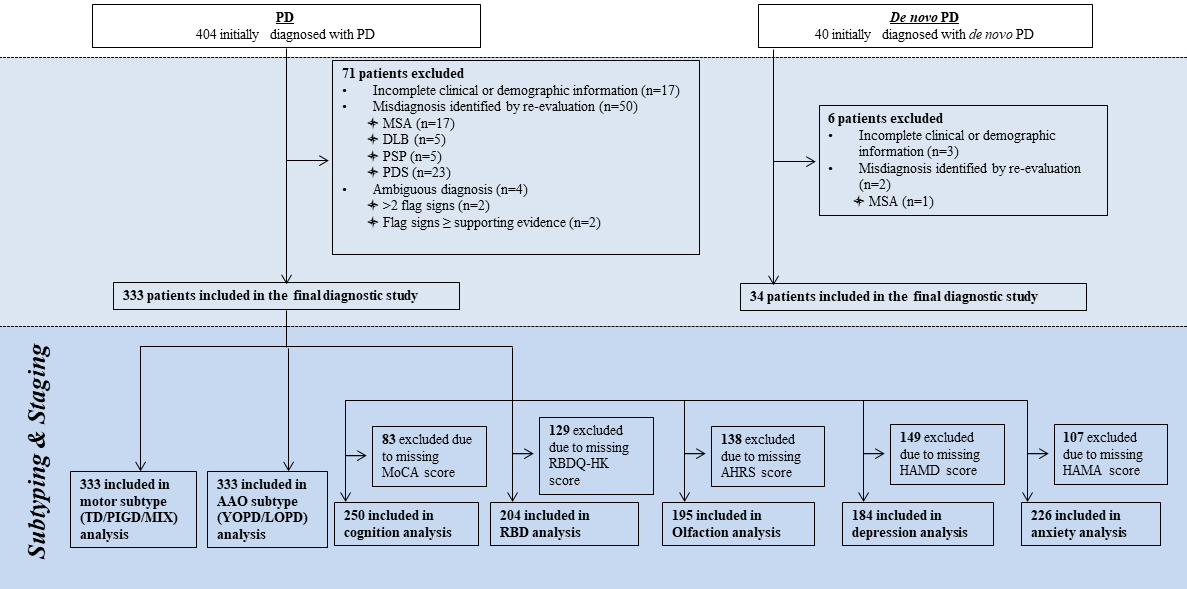

Supplement: Supplementary file 1 [file Image_1.tif]

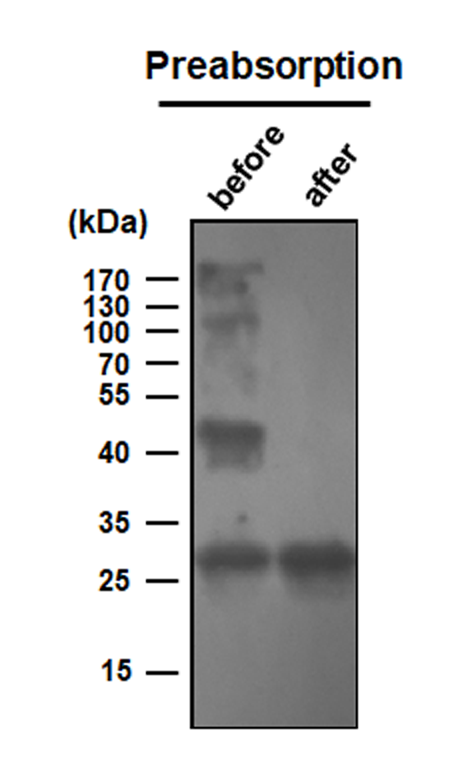

Supplement: Supplementary file 2 [file Image_2.tif]

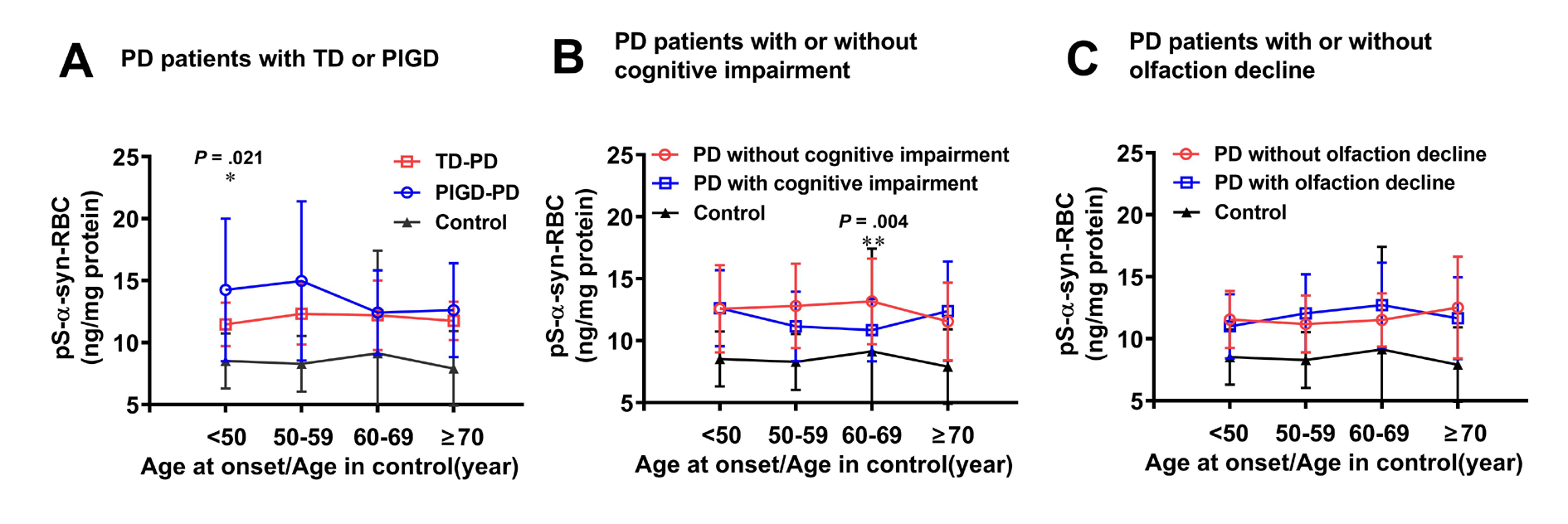

Supplement: Supplementary file 3 [file Image_3.tif]
