# Supplementary material for: Alterations of Erythrocytic Phosphorylated Alpha-Synuclein in Different Subtypes and Stages of Parkinson's Disease
Source: Front Aging Neurosci. 2021 Sep 29;13:623977. doi: 10.3389/fnagi.2021.623977 (PMC8511781; doi:10.3389/fnagi.2021.623977)
Supplement: Supplementary file 4 [file Data_Sheet_1.pdf]

SUPPLEMENTARY METHODS

Scales for quantifying the symptoms of Parkinson's disease (PD)

Movement Disorder Society–sponsored revision of the Unified Parkinson's Disease Rating Scale ratings part III (MDS-UPDRS III) and Hoehn & Yahr (H & Y) stage for motor symptoms; Mini-Mental State Examination (MMSE) scores and Montreal Cognitive Assessment (MoCA) scores for cognitive function; Hyposmia rating scale (AHRS) scores for olfactory function; Rapid eye movement (REM) sleep behavior disorder questionnaire-Hong Kong (RBDQ-HK) scores for sleep disorder; Hamilton Depression Scale (HAMD) and Hamilton Anxiety Scale (HAMA) for neuropsychological symptoms.

Definition of motor and non-motor subtypes of PD

Younger age at onset was defined as onset of motor symptoms before the age of 50 years. Motor subtypes of PD were classified as manifesting the tremor-dominant (TD), postural instability and gait difficulty (PIGD) or mixed (MIX) phenotype, according to the ratio of mean tremor score/mean PIGD score in MDS-UPDRS, as described previously (Jankovic et al., 1990). Non-motor subtypes were defined by the existence of cognitive impairment, neuropsychiatric symptoms, sleep disorders and olfactory decline, according to cut-off points on the scores quantifying these symptoms: MMSE and MoCA for cognitive impairment, AHRS for olfactory loss, RBDQ-HK for RBD, HAMD for depression and HAMA for anxiety.

Production and purification of recombinant α-syn

A recombinant α-synuclein (α-syn) monomer (wild type α-syn) was prepared as previously described (Alim et al., 2004). Briefly, the pET-15b-NACP plasmids expressing human α-syn were transformed into Escherichia coli BL21 cells, and the α-syn proteins expressed were purified using sequential ion exchange, hydrophobic and reverse phase chromatography. Purity of the protein was determined by Coomassie Brilliant Blue staining and immunoblot analysis.

Preparation of phosphorylated α-syn

Serine129-phosphorylated α-syn (pS-α-syn) was prepared from recombinant human α-syn as previously described (Sasakawa et al., 2007; Mathiesen et al., 2018). Purified α-syn was first incubated with casein kinase II (New England Biolabs, Ipswich, MA, USA), and the resultant pS129 α-syn was purified by anion exchange chromatography and verified by immunoblotting with a rabbit anti-pS-α-syn antibody combined with mass spectrometry. The pS-α-syn was concentrated by ammonium sulfate precipitation. Purity of the protein was determined by Coomassie Brilliant Blue staining and immunoblot analysis.

Spike-and-recovery and linearity-of-dilution assessments

Briefly, after 100× dilution with 0.01M PBS, a total of 18 samples of isolated RBCs were mixed pairwise to obtain 9 sample matrix. Different concentrations (spikes) (0μg/ml for no, 1.5μg/ml for low, 3μg/ml for medium and 4.5μg/ml for high) of standard pS-α-syn samples were added to each sample (in triplicate) and measured their absorbances. The responses of the sample matrix and standard diluent were compared based on values calculated from a standard curve. The recovery rate for each spike level was calculated using the following formula: observed concentration/expected concentration ×100%.

REFERENCES

Jankovic, J., McDermott, M., Carter, J., Gauthier, S., Goetz, C., Golbe, L., et al. (1990). Variable expression of Parkinson's disease: a base-line analysis of the DATATOP cohort. The Parkinson Study Group. *Neurology* 40, 1529–1534. doi: 10.1212/wnl.40.10.1529

Alim, M. A., Ma, Q. L., Takeda, K., Aizawa, T., Matsubara, M., Nakamura, M., et al. (2004). Demonstration of a role for alpha-synuclein as a functional microtubule-associated protein. *J. Alzheimers. Dis.* 6, 435–442. doi: 10.3233/jad-2004-6412

Sasakawa, H., Sakata, E., Yamaguchi, Y., Masuda, M., Mori, T., Kurimoto, E., et al. (2007). Ultra-high field NMRstudies of antibody binding and site-specific phosphorylation of α-synuclein. *Biochem. Biophys. Res. Commun.* 363, 795–799. doi: 10.1016/j.bbrc.2007.09.048

Mathiesen, R., Chriél, M., Struve, T., and Heegaard, P. M. H. (2018). Quantitative immunoassay for mink immunoglobulin in serum and milk. *Acta. Vet. Scand.* 60, 36. doi: 10.1186/s13028-018-0391-7

SUPPLEMENTARY FIGURE LEGENDS

Figure S1. Flow chart of the cohort study.

Figure S2. Western blot analysis of the specificity of antibodies used for ELISA detection of pS-α-syn in RBC lysates

RBC lysates before and after preabsorbed by Santa Cruz pS-α-syn antibody were detected by a different anti-pS-α-syn antibody (Wako). After absorption, the α-syn oligomers more than 40 kDa were disappeared. The band around 28 kDa, which is the hemoglobin dimer, was not absorbed.

Figure S3. Differences of pS-α-syn-RBC levels between patients with PD subtypes and ages at onset (AAOs)

(A) PD patients with TD (*n* = 50) vs with PIGD (*n* = 103); **(B)** PD patients with (*n* =66) vs without (*n* = 184) cognitive impairment; **(C)** PD patients with (*n* = 56) vs without (*n* = 139) olfactory decline. TD: tremor-dominant; PIGD: postural instability and gait difficulty. Error bar: Mean ± SD.

SUPPLEMENTARY TABLES

Table S1 ELISA spike and recovery assessments of pS-α-syn in RBC lysates

Samples were assayed by adding 100 µl of sample and different spike stock solution to yield the intended 0, 1.5, 3 or 4.5 µg/ml spike concentration. Values reported for spike samples reflect subtraction of the endogenous (No spike) values. Recoveries for spiked test samples were calculated by comparison to the measured recovery of spiked diluent. All values represent the average of three replications.

Sample	No spike （0 µg/ml）	Low spike （1.5 µg/ml）	Medium spike （3.0 µg/ml）	High spike （4.5 µg/ml）
0.01M PBS	0	1.70	2.80	4.00
RBC1	1.35	1.45	2.38	3.81
RBC2	1.13	1.56	2.87	3.65
RBC3	2.07	1.41	2.57	3.98
RBC4	1.64	1.59	2.39	3.82
RBC5	1.91	1.46	2.48	3.96
RBC6	2.23	1.48	2.46	3.83
RBC7	1.86	1.51	2.72	3.75
RBC8	1.77	1.49	2.59	3.77
RBC9	1.42	1.47	2.36	3.98

Table S2 Typical presentation for summarizing spike and recovery results

Sample (n)	Spike Level	Expected	Observed	Recovery (%)
	Low (1.5 µg/ml)	1.7	1.49	87.7
RBC (9)	Med (3.0 µg/ml)	2.8	2.54	90.7
	High (4.5 µg/ml)	4.0	4.27	95.9

Table S3 Tested values of blank samples

**Sample No**	1	2	3	4	5	6	7	8	9	10
**Values (µg/mL)**	0.45	0.54	0.52	0.53	0.54	0.53	0.52	0.55	0.49	0.51
**Sample No**	11	12	13	14	15	16	17	18	19	20
**Values (µg/mL)**	0.47	0.55	0.76	0.58	0.57	0.56	0.56	0.58	0.60	0.58

Table S4 Lower limit of detection (LLOD) and lower limit of quantification (LLOQ) of blank samples

Items	LLOD	LLOQ
Test values (µg/ml)	0.18	0.60

Table S5 Overall and clinical variable-stratified comparisons of pS-α-syn-RBC levels between non-motor subtypes of Parkinson's disease

Scale	Depression			Anxiety		
	**No (*n*=62)**	**Yes (*n*=122)**	***p*-Value**	**No (*n*=56)**	**Yes (*n*=170)**	***p*-Value**
Sex (Male / Female)	36/26	60/62	-	26/27	53/54	-
Age	61.81±11.83	61.54±9.01	-	60.70±10.40	62.50±8.88	-
Age at onset	57.08±14.25	56.11±10.58	-	56.38±10.49	56.82±12.17	-
Disease duration, y	3.81±3.01	5.31±4.87	0.153	4.35±3.96	5.30±4.94	0.069
LEDD, mg/day	325.38±333.85	425.08±399.84	0.353	330.92±274.39	435.95±368.80	0.167
H & Y stage, median	2 (2, 3)	2 (2, 3)	-	2 (2, 3)	2 (2, 3)	-
pS-α-syn-RBC levels	12.16±2.74	11.69±2.98	0.147	12.02±2.71	11.66±2.91	0.146
**pS-α-syn-RBC levels in patients stratified by age at onset, ng/mg [a]**						
< 50	10.30±1.68	12.00±2.95	0.232	10.79±2.29	11.71±2.58	0.724
50-59	12.47±2.63	11.04±2.67	0.181	11.60±2.18	11.68±2.92	0.810
60-69	12.88±2.96	11.99±3.26	0.680	12.68±2.57	11.74±3.07	0.717
≥ 70	10.81±2.49	12.76±3.69	0.176	15.66±4.89	11.32±3.21	0.171
**pS-α-syn-RBC levels in patients stratified by disease duration, ng/mg [b]**						
0-2	12.21±3.19	11.93±3.25	0.258	12.34±3.00	12.18±3.43	0.246
3-5	12.11±2.69	11.58±3.17	0.278	12.12±2.88	11.35±2.45	0.596
5-10	11.55±2.18	10.97±2.40	0.873	11.17±1.70	10.87±2.56	0.562
> 10	10.55±0.48	12.69±3.24	0.317	11.88±2.60	12.47±2.54	0.941
**pS-α-syn-RBC levels in patients stratified by H&Y stages, ng/mg [c]**						
1	10.68±3.01	12.17±3.21	0.518	10.64±2.25	11.88±2.93	0.273
2	12.09±2.52	11.46±2.83	0.214	12.46±2.74	11.26±2.56	0.115
3	12.52±3.16	11.75±3.26	0.589	12.02±2.81	11.96±3.28	0.899
4-5	12.44±3.30	11.80±2.74	0.757	12.44±3.30	11.80±2.75	0.757

[a, c] Covariance analysis adjusting for sex, disease duration and LEDD; [b] Covariance analysis adjusting for sex, age and LEDD. For cognitive decline, MoCA score cut-offs of 17 (illiteracy), 20 (primary school education), 23 (secondary school education) of 30 points; for RBD, a cut-off of 19 on the RBDQ-HK score; for olfactory loss, a cut-off of 22 on the AHRS score; for depression, a cut-off of 8 on the HAMD score; for anxiety, a cut-off of 7 on the HAMA.
